# Supplementary material for: Kathon Induces Fibrotic Inflammation in Lungs: The First Animal Study Revealing a Causal Relationship between Humidifier Disinfectant Exposure and Eosinophil and Th2-Mediated Fibrosis Induction
Source: Molecules. 2020 Oct 14;25(20):4684. doi: 10.3390/molecules25204684 (PMC7587358; doi:10.3390/molecules25204684)
Supplement: Supplementary file 1 [file molecules-25-04684-s001.pdf]

Supporting Information for

Kathon induces fibrotic inflammation in lungs: The first animal study revealing a causal relationship between humidifier disinfectant exposure and eosinophil and Th2-mediated fibrosis induction

Mi-Kyung Song [1,2], Dong Im Kim [1] and Kyuhong Lee [1,2,*]

[1] *National Center for Efficacy Evaluation of Respiratory Disease product, Korea Institute of Toxicology, 30 Baehak1-gil, Jeongeup, Jeollabuk-do 56212, Republic of Korea,* [2] *Department of Human and Environmental Toxicology, University of Science & Technology, Daejeon 34113, Republic of Korea*

Correspondence: *Kyuhong Lee, National Center for Efficacy Evaluation of Respiratory Disease Product, Korea Institute of Toxicology, 30 Baehak1-gil, Jeongeup, Jeollabuk-do 56212, Republic of Korea. Fax: 082-63-570-8797; E-mail:* khleekit@gmail.com

Table S1. Quantitative histopathological assessment of the lung sections.

		Naive Control	Saline	Kathon (mg/kg)	
				**0.57**	**1.14**
	No. of animals	5	5	4	5
Infiltrate, eosinophilic cells, mainly perivascular	Total Number of affected	0	0	2	5
	Slight	0	0	2	0
	Moderate	0	0	0	2
	Severe	0	0	0	3
	Scoring	**0.00**	**0.00**	**1.00***	**3.60**** **
Granulomatous inflammation, alveolar	Total Number of affected	0	0	4	5
	Minimal	0	0	1	0
	Slight	0	0	0	0
	Moderate	0	0	3	3
	Severe	0	0	0	2
	Scoring	**0.00**	**0.00**	**2.50******	**3.40******
Pulmonary fibrosis, alveolar duct/alveolar wall	Total Number of affected	0	0	2	5
	Minimal	0	0	2	2
	Slight	0	0	0	3
	Scoring	**0.00**	**0.00**	**0.50**	**1.60******
Inflammatory cell infiltrate, mixed, mononuclear/lymphocytic cell	Total Number of affected	0	0	3	5
	Minimal	0	0	0	0
	Slight	0	0	3	3
	Moderate	0	0	0	2
	Scoring	**0.00**	**0.00**	**1.50****	**2.40******
Hyperplasia, mucous cell, mainly bronchiole	Total Number of affected	0	5	4	5
	Minimal	0	0	1	0
	Slight	0	1	2	3
	Moderate	0	4	1	2
	Scoring	**0.00**	**0.00**	**2.00******	**2.40******

Table S2 : RNA QC results

**Sample ID**	**ng/µl**	**260/280**	**RIN**
Control-1	429.1	1.90	8.1
Control-2	449.1	1.89	7.5
Control-3	720.2	2.04	8.4
Kathon 1.14 mg/kg-1	3423.7	2.01	9.1
Kathon 1.14 mg/kg-2	1785.3	2.13	8.9
Kathon 1.14 mg/kg-3	2001.3	2.13	8.8

SUPPORTING FIGURE LEGENDS

Figure S1. Schematic diagram of the experimental protocol.

Figure S1.

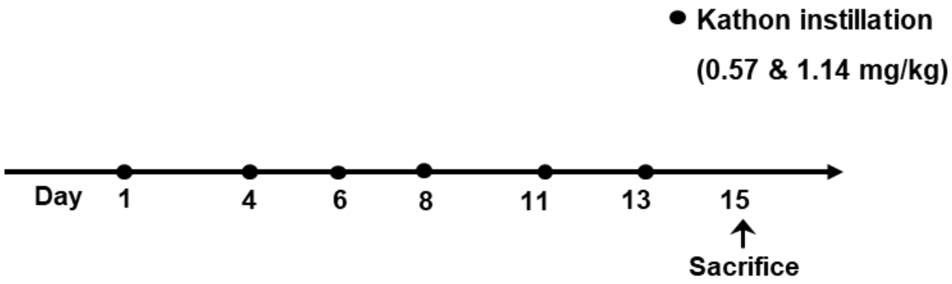
Kathon instillation
(0.57 & 1.14 mg/kg)
Day
1
4
6
8
11
13
15
Sacrifice
